# Asymmetric effects of amphipathic molecules on mechanosensitive channels

**DOI:** 10.1038/s41598-022-14446-w

**Published:** 2022-06-15

**Authors:** Omid Bavi, Zijing Zhou, Navid Bavi, S. Mehdi Vaez Allaei, Charles D. Cox, B. Martinac

**Affiliations:** 1 Department of Mechanical and Aerospace Engineering, Shiraz University of Technology, Shiraz, Iran; 2 Molecular Cardiology and Biophysics Division, Victor Chang Cardiac Research Institute, Darlinghurst, NSW 2010 Australia; 3 Department of Biochemistry and Molecular Biology, The University of Chicago, Chicago, IL USA; 4 Department of Physics, University of Tehran, 1439955961 Tehran, Iran; 5 Faculty of Medicine, St Vincent’s Clinical School, University of New South Wales, Darlinghurst, NSW 2010 Australia

**Keywords:** Computational biophysics, Mathematics and computing

## Abstract

Mechanosensitive (MS) ion channels are primary transducers of mechanical force into electrical and/or chemical intracellular signals. Many diverse MS channel families have been shown to respond to membrane forces. As a result of this intimate relationship with the membrane and proximal lipids, amphipathic compounds exert significant effects on the gating of MS channels. Here, we performed all-atom molecular dynamics (MD) simulations and employed patch-clamp recording to investigate the effect of two amphipaths, Fluorouracil (5-FU) a chemotherapy agent, and the anaesthetic trifluoroethanol (TFE) on structurally distinct mechanosensitive channels. We show that these amphipaths have a profound effect on the bilayer order parameter as well as transbilayer pressure profile. We used bacterial mechanosensitive channels (MscL/MscS) and a eukaryotic mechanosensitive channel (TREK-1) as force-from-lipids reporters and showed that these amphipaths have differential effects on these channels depending on the amphipaths’ size and shape as well as which leaflet of the bilayer they incorporate into. 5-FU is more asymmetric in shape and size than TFE and does not penetrate as deep within the bilayer as TFE. Thereby, 5-FU has a more profound effect on the bilayer and channel activity than TFE at much lower concentrations. We postulate that asymmetric effects of amphipathic molecules on mechanosensitive membrane proteins through the bilayer represents a general regulatory mechanism for these proteins.

## Introduction

Bacterial mechanosensitive (MS) channels MscS and MscL have been at the forefront of MS channel research since their discovery^1^. They continue to serve as excellent molecular models for studies of the basic biophysical principles underlying mechanotransduction processes using molecular dynamics simulations^2^, functional assays^3–5^ and cryo-EM techniques^6,7^. They were the first MS channels shown to be inherently mechanosensitive and gated by membrane tension caused by an increase of turgor pressure in bacterial cells^8,9^. They were also instrumental in establishing the “force-from-lipids” principle^10,11^ as the unifying and evolutionarily conserved biophysical principle controlling gating of eukaryotic MS ion channels, including Piezo1^12,13^, TREK-1, TREK-2 and TRAAK^14,15^ as well as OSCA/TMEM63^16^.

The “gold standard” method to study how mechanical force is converted into structural changes in MS channels is reconstitution of MS channels into liposomes^17–19^. Combined with electrophysiological investigative techniques, this reductionist approach showed that these channels could be driven over their full dynamic range in the presence of only lipids^20^. As a result, there is a close relationship between the physico-chemical properties of the lipid bilayer and structural dynamics of these channels^5,19,21,22^. Such observations began with the use of amphipathic compounds that were shown to activate MscS and MscL when inserted into one of the leaflets of the membrane bilayer^23^. Lysophosphatidylcholine (LPC) is one of the amphipaths that has been widely used for studies of gating and structural dynamics of both channels^19,24–26^. Other examples of compounds with amphipathic properties used in MS channel studies include local anaesthetics^10^ and polyunsaturated fatty acids (PUFAs)^27,28^. In fact, in many cases the effect of these molecules extends even beyond MS channels to other membrane embedded proteins^29–31^. The mechanism underlying these effects on membrane proteins are still not completely understood.

In addition, another motivation for this study arises from the significant effort and interest invested in making cancer drugs less invasive and more effective. One of the very promising methods towards this goal has been provided by the development of liposomal drug delivery systems^32,33^. As one of the most extensively studied and advanced drug delivery systems, liposomes provide a widely applicable method of drug encapsulation and administration. They present an effective non-toxic method for administration of highly toxic chemotherapeutic drugs, such as anthracyclines doxorubicin and daunorubicin^34^ or antifungal agents, such as amphotericin^35^. Using this method, drugs are encapsulated inside the liposomes and are released via different actuators such as membrane-embedded nanoparticles or proteins^36,37^. The most promising liposome delivery systems are “stimulus-triggered” liposomes. Among these, pH-triggered liposomes are intensely investigated liposomal preparations^38–41^. Caveats of this technology result from possible drug interactions with the embedded proteins and/or with their surrounding lipid membrane which may reduce efficiency of the drugs. Although drug-protein interactions are well known, the biological ramifications of drug-membrane interactions are less clear.

Here we used two amphipathic compounds, a cancer chemotherapeutic 5-Fluorouracil (5-FU) and the anaesthetic trifluoroethanol (TFE). 5-FU is a pyrimidine analogue, which is an antineoplastic antimetabolite used for multiple cancers^36^. TFE is a key precursor for the inhaled anaesthetic isoflurane but itself is not used as an anaesthetic because of its toxicity and is therefore, predominantly used as a solvent in organic chemistry^37^. To study the 5-FU and TFE effect on MscL and its lipid surrounding we have combined all-atom MD simulations with patch clamp recording. To demonstrate the universality of the amphipathic drug effects on MS channels we show how 5-FU and TFE-induced surface tension perturbations affect MscL, MscS and TREK-1 channels. Our findings have implications for the development of drug-carrying liposomes when considering drug storage, transport, and release when selecting optimal liposomal carriers in terms of their physicochemical properties.

## Materials and methods

### Molecular dynamics simulations

All molecular dynamic simulations were performed using NAMD 2–10 package and CHARMM36 parameters as the force field^42^. Visual Molecular Dynamics (VMD) and PYMOL were used for all visualizations^43^. The equilibration steps have been done identically to our previous MD simulation of *E. coli* MscL (EcMscL) in which the 3D structure of MscL channel was generated based on a homology model adopted from *Mycobacterium tuberculosis* MscL (Mt-MscL, PDB: 2OAR)^2^. The resultant MscL models were embedded into a 1-palmitoyl-2-oleoyl-sn-glycero-3-phospho-ethanolamine (POPE) bilayer comprised of 222 lipids. The lipid heads and tails were in turn randomised and equilibrated for ~ 1 ns at 298 K, while the rest of the system was fixed. Then the protein and lipids were solvated in a 120 × 120 × 130–Å water box. After randomization of the POPE lipid tails (1 ns), the whole system was equilibrated for 62 ns with a time step of 2 fs with no restraints. Then we added 2% volume to volume ratio (v/v) Fluorouracil (5-FU) (C4H3FN2O2) molecules into one side of the bilayer. The force field parameters for 5-FU was obtained from CHARMM GUI^44^. By temporal restraining of the protein backbone with a harmonic spring constant of 1 kcal mol^−1^ Å^−2^, the drug molecules were allowed to equilibrate in the system for 15 ns. Then we released the protein restraints and continued the simulation for another 135 ns to monitor the effect of 5-FU on the channel activity. We used the MEMBPLUGIN S_CD_ Order Parameter Tool^45^ to compute the S_CD_ (deuterium order parameters) of the lipid acyl chains. Also, we used a custom-written tcl code for calculating the pressure profiles and thickness of lipids as well as changes in channel configuration.

### Protein purification and liposome reconstitution

The purification of the 6xHis-tagged MscL G22S protein was carried out as previously described^46^. Briefly, G22S mutant MscL was expressed in *E. coli* BL21(DE3) cells (Novagen), grown at 37 °C in lysogeny broth to an OD600 of ∼ 0.6, and then induced with 1 mM IPTG for 3 h. Subsequently the cells were centrifuged and the pellet was resuspended in phosphate-buffered saline (PBS; 10 mM Na_2_HPO_4_, 1.8 mM KH_2_PO_4_, pH 7.5, 137 mM NaCl, 2.7 mM KCl) with ∼ 0.01 mg/mL DNase (Sigma DN25) and 0.02% (wt/vol) phenylmethylsulfonyl fluoride (Amresco M145), and the cells were broken with a TS5/48/AE/6 A cell disrupter (Constant Systems) at 31,000 psi at 4 °C. Cell debris was removed by centrifugation (12,000 × g for 15 min at 4 °C), and membranes were then pelleted at 45,000 rpm in a Type 45 Ti rotor (Beckman) for 3 h at 4 °C. Membrane pellets were solubilized overnight at 4 °C with 8 mM DDM in PBS, pH 7.5. After centrifugation at 12,000 × g for 20 min at 4 °C, the supernatant was applied to cobalt Sepharose (Talon, 635,502, Clontech). The column was washed three times with 40 mL 35 mM imidazole in PBS, pH 7.5, and protein was eluted using 500 mM imidazole in PBS, pH 7.5. The imidazole concentration was decreased using a 100-kDa Amicon-15 centrifugal filter unit (Merck Millipore) with 1 mM DDM in PBS, pH 7.5. Liposome reconstitution of the MscL-G22S was carried out using the modified method previously published^47^. L-α-Phosphatidylcholine from soy extract polar phospholipids (SPL) (41602P, Avanti) and POPE (850757P, Avanti) were used at the indicative ratio for liposome preparation. Purified MscL-G22S was reconstituted and co-reconstituted with MscS into the lipid bilayers at 1:2000 (w/w) ratio. The proteolipid mixture was dehydrated in a desiccator overnight at room temperature, then rehydrated with the solution containing 200 mM KCl and 5 mM HEPES (pH 7.2) at 4 °C for 3 h.

### Cell culture and transfection

HEK293T cells (ATCC) were transiently transfected with TREK-1 and were used for recording the channel activity from cell-attached or excised inside-out patches. The TREK-1 construct was a gift from Dr Philip Gottlieb (SUNY Buffalo). HEK293T were maintained in DMEM containing 10% fetal bovine serum (Gibco). Transient transfections were performed using Lipofectamine as per manufacturer’s instructions and using 0.5–1 µg plasmid per 35 mm culture dishes. Cells were used for patch clamping 16–24 h post transfection.

### Patch clamp recording

All recordings from MscL-G22S and MscS were performed in symmetrical bath and pipette solution containing 200 mM KCl, 40 mM MgCl_2_, 5 mM HEPES (pH 7.2) (recording buffer). 2,2,2-Trifluoroethanol (TFE, Sigma) was added to the recording chamber at 1:200 (v/v) ratio to recording buffer. Fluorouracil (5-FU, Sigma) for MscL-G22S experiments was dissolved with DMSO to 0.5 M, then diluted to 5 or 10 mM with the recording buffer. The diluted reagent was then added to the recording chamber at 1:20 (v/v) ratio to recording buffer to achieve a final concentration of 250 or 500 µM, respectively. DMSO (Sigma) was 50-fold diluted with the recording buffer, then added to the recording chamber at 1:20 (v/v) ratio to recording buffer. All reagents were added upon forming of the giga-seal.

The experimental solutions for the TREK-1 channel recording contained 140 mM NaCl, 3 mM KCl, 1 mM MgCl_2_, 1 mM CaCl_2_, 10 mM glucose, 10 mM HEPES (pH 7.2) as extracellular buffer, and 90 mM K-Asp, 50 mM KCl, 1 mM MgCl_2_, 10 mM glucose, 10 mM HEPES (pH 7.2) as intercellular buffer. Fluorouracil (5-FU, Sigma) was dissolved with DMSO to 0.5 M, then diluted to 5 mM with the recording buffer. The diluted reagent was then added to the recording chamber at 1:20 (v/v) ratio to recording buffer to achieve a final concentration of 250 µM. DMSO (Sigma) was 100-fold diluted with the recording buffer, then added to the recording chamber at 1:20 (v/v) ratio. All reagents were added upon forming of the giga-seal.

Borosilicate glass pipettes (Drummond Scientific, Broomall, PA) were pulled using a Narishige puller (PP-83; Narishige) to achieve a pipette resistance at 2–3 MΩ. The channel currents were recorded with an AxoPatch 1D amplifier (Axon Instruments) in the inside-out patch configuration, and data were taken at 5-kHz sampling rate with 2-kHz filtration. Negative pressure was applied using a high-speed pressure clamp-1 apparatus (HSPC-1; ALA Scientific Instruments).

The mid-maximum pressure (P_1/2_) refers to the pressure required to reach half of the maximum current. The channel activity vs pressure was fitted to a Boltzmann function relating the current (I) to pressure (P) traces. For generating normalized peak current (Normalized I_max_), peak currents before or after applying the reagents were recorded using the pClamp10 software. The peak currents after treatment were divided by that before treatment from the same individual patch. n and P values are indicated in the figure legends for individual experiments.

### Preparation of giant *E. coli* spheroplasts

Both wild-type MscL and MscS were expressed in *E. coli* MJF612 (ΔmscL::cm, ΔmscS, ΔmscK::kan,ΔybdG::apr) cells using a pETDuet-1 vector^2^ and spheroplasts were prepared using a standardized protocol^18,48^. Briefly, MJF612 cells were grown for 1–2 h in the presence of cephalexin (final concentration 60 μg ml^−1^) to form *E. coli* snakes (length—50 to 150 μm). Cells were then digested for 3–7 min in the presence of 0.8 M sucrose, 60 mM TRIS, pH 7.2, lysozyme (final concentration 0.2 mg ml^−1^) and EDTA (6.3 mM). A stop solution (0.8 M sucrose, 20 mM MgCl_2_, 60 mM TRIS pH 7.2) was added, and the spheroplasts were collected by centrifugation.

### Statistics

All data are expressed as means ± SEM; n represents the number of independent biological replicates. Unless indicated otherwise, Student’s two-tailed t test was used for statistical analysis, and *P* < 0.05 was considered statistically significant.

## Results

**Figure 1:**
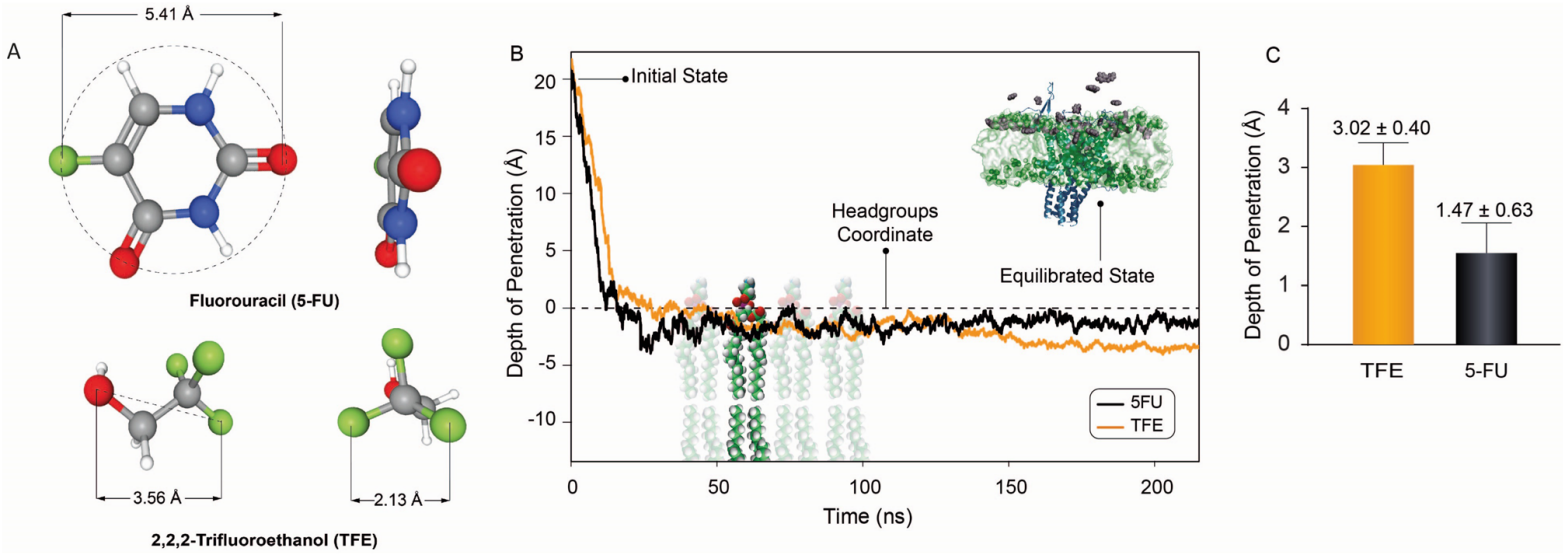
Schematics of 5-Fu and TFE molecules and their interaction with a POPE membrane (based on their equilibrium state). (**A**) timeline of the drug’s diffusion into lipid bilayer (**B**), and averaged depth of the penetration over the simulation time (**C**). Inset shows the adsorption of 5-FU (grey) into a POPE lipid bilayer (green) containing MscL (cyan). The phosphate atoms of the bilayer have been represented as grey spheres to better visualize the lipid-water interface. As it can be seen from (**A**), the 5-FU molecule is completely planner. The Topological Polar Surface Area of 5-FU is 58.2 Å^2^, while this value has been reported 20.2 Å^2^ for TFE molecules. (**C**) The mean penetration depths of TFE and 5-FU molecules are − 3.02 ± 0.40 Å and − 1.47 ± 0.63, Å respectively (mean ± SD, the changes are statistically significant; *P* < 0.05; Student’s t-test was performed).

**Figure 2:**
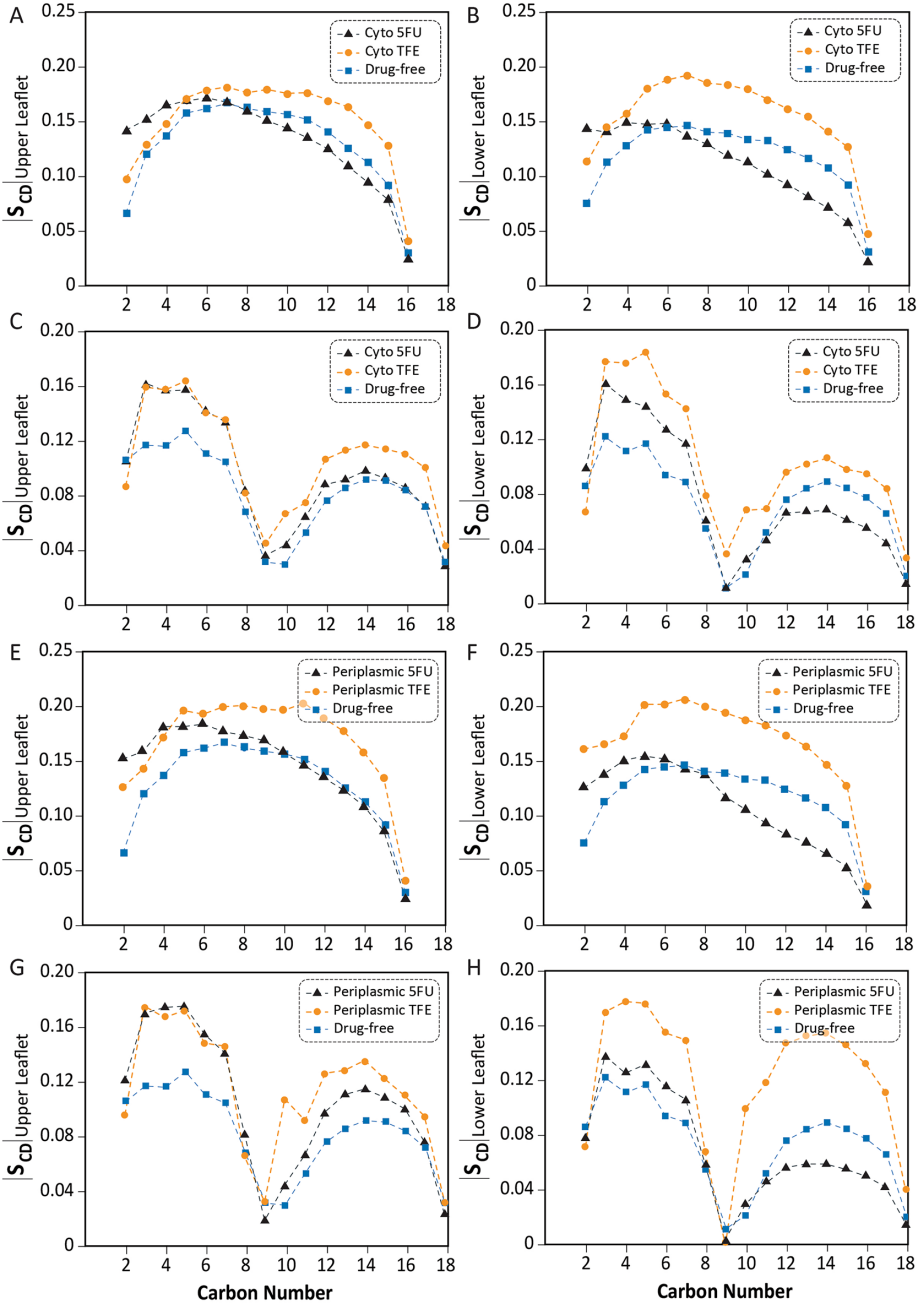
Effect of 5-FU and TFE adsorption to the bilayer from the cytoplasmic and periplasmic sides. Sn-1 order parameter (**A**, **B**, **E**, **F**) and Sn-2 order parameter (**C**, **D**, **G**, **H**) of a POPE bilayer containing MscL are affected by both amphipaths. Effect of the drugs on Sn-1 and Sn-2 when added to the cytoplasmic side (**A**–**D**) and periplasmic side (**E**–**H**). The vertical axes represent the absolute values of calculated deuterium order parameters which show that TFE molecules interact with the lipid molecules leading to an increase of the order parameters. While the presence of 5-FU increases the order parameter for carbon atoms located close to the lipid/water interface its presence decreases the order parameter for those in the bilayer center. Data represents mean ± SD of 3 replicate simulations using 2% v/v TFE and 25 mM 5-FU.

**Figure 3:**
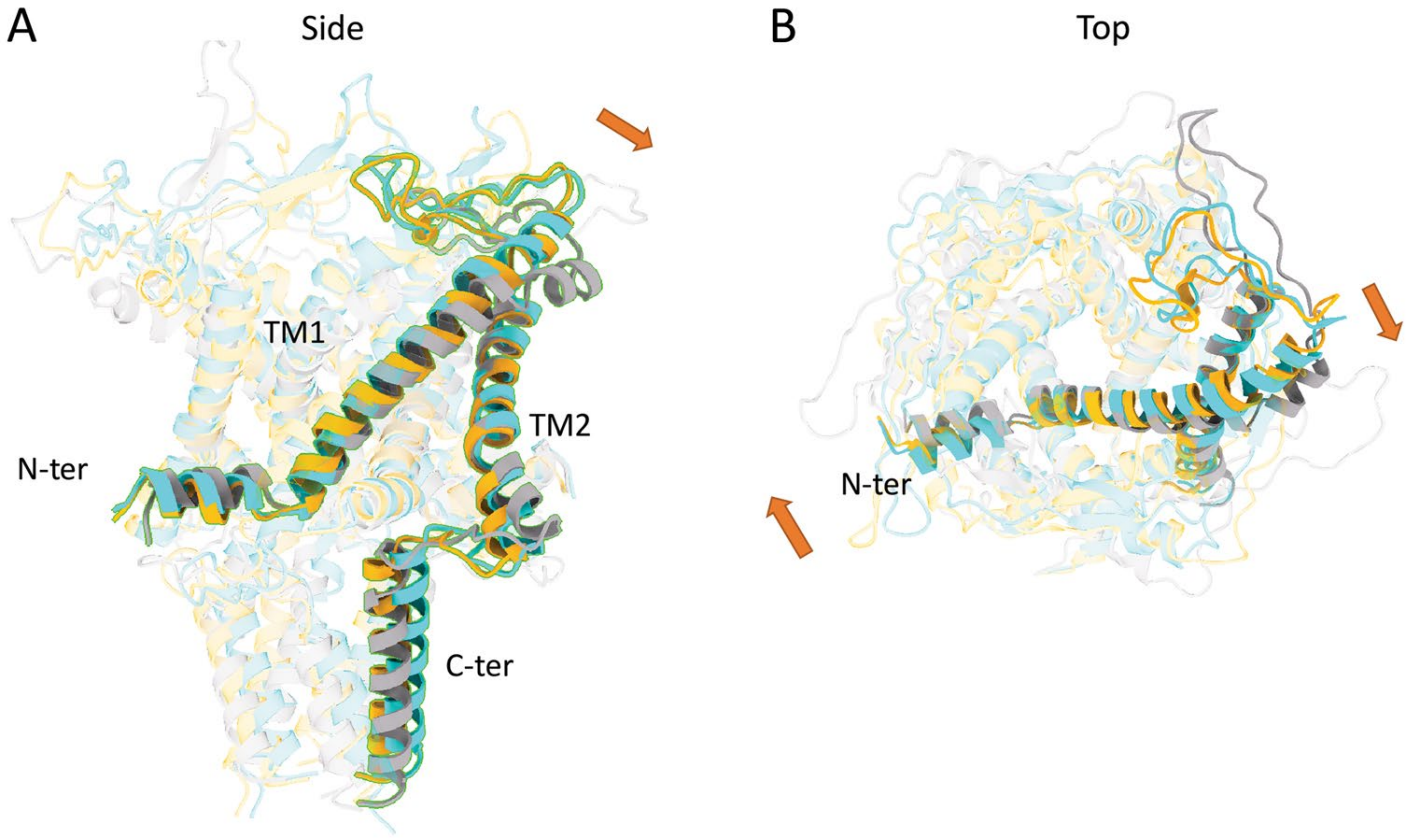
Structural rearrangement of MscL helices due to 5-FU and TFE insertion using MD simulations. Red arrows show the parts of the channel that are moving due to the presence of the drugs. Cyan WT (not treated), Grey is 5FU and Orange is TFE.

The effect of 5-FU and TFE on bilayer permeation, bilayer thickness, transbilayer pressure profile (also referred to as lateral pressure profile), order parameter and channel conformational changes have been examined using all-atom MD simulation (Figs. 1, 2, 3). As demonstrated in Fig. 1, TFE molecules were adsorbed more readily and reside deeper within the bilayer (below the phosphate group) compared to the 5-FU molecules while being ~ 1.5 times smaller in size and molecular weight. Yet like 5-FU, TFE molecules cannot completely penetrate the hydrophobic barrier. This result suggests high adsorption of 5-FU by lipids such as POPE.

The influence of directionality of the drug was tested with a bilayer system containing MscL by adding the drugs from the cytoplasmic/lower leaflet and periplasmic/upper leaflet sides as per the MscL channel orientation (Fig. 2). Interestingly, the presence of TFE molecules in both the cyto- and periplasmic leaflets increases the order of the hydrocarbon chains of each of the upper and lower bilayer membranes in the Sn-1 and Sn-2 branches. These effects are more pronounced toward the bilayer mid-plane. This increase in order is due to the significant penetration of molecules deep into the membrane, which will increase the lipid thickness. The situation is different with FU molecules. The presence of FU molecules inside and outside the cell increases the order parameter in the upper leaflet while decreasing it in the lower leaflet (specifically at the end of the hydrocarbon chains: carbon atoms number eight and under). This difference is due to physicochemical properties of the molecule and the depth of penetration of these molecules into the membrane. These factors are also directly influenced by the asymmetry of the protein channel shape in the membrane and its effect on the order of hydrocarbon chains around the channel. In general, it is known that the presence of a membrane protein will affect membrane properties including the order parameter and depending on the hydrophobic length of the protein the thickness of the membrane^49–51^. The presence of amphipathic drugs can neutralize or exacerbate these effects.

To investigate whether these drugs could also directly interact with the protein, we mapped the drug-protein interaction energy on the protein for both 5-FU and TFE. As shown in SI Fig. 1 and SI Table 1, there is also a direct interaction (6.84 kcal/mol for 5-FU and 0.63 kcal/mol for TFE) with the protein for both molecules. 5FU, given its higher charge density than TFE, interacts stronger with the protein. Nevertheless, these interactions are energetically small compared to the major effects they have on the lipid bilayer mechanics.

We did not observe any insertion of the amphipaths into lipid filled pockets or grooves in MscL that have been noted in the structures of many MS channels^2,6,52–54^, regardless of which side the drug was added from. However, given computational limitations we cannot reach the experimental timescales in our simulations, hence we cannot completely exclude this possibility. The residues with the highest interaction with the TFE are LYS55 (− 0.63 kcal/mol) and ASP39 residues which also interact more strongly with the 5FU molecules (− 6.84 kcal/mol) (SI Table 1).

We also probed the effect of 5-FU at 3 different concentrations using all-atom MD simulations (SI Fig. 2). As the concentration of 5-FU increases, the Sn-1 order parameter for the upper leaflet (the side containing the drug) increases while the Sn-2 parameter decreases. For the lower leaflet, the Sn-1 parameter decreases with boosting the 5-FU concentration, while the Sn-2 parameter remains almost unchanged.

Moreover, 5-FU greatly influences the lateral pressure profile of the bilayer (SI Fig. 3). The area under the lateral pressure profile is almost two-fold larger once the drug is incorporated into the bilayer. This could be due to the size, shape, and hydrophobicity of 5-FU. In addition, the difference between the pressure in upper and lower leaflets of the bilayer can induce a local curvature, which can on its own modulate the activity of the embedded MscL channel^55,56^. Albeit accurate quantification of such local curvature indeed requires systems with much larger dimensions than ours to be unaffected by the periodic boundary conditions. From our simulations, it is evident that in the absence of any external force, the effect of 5-FU on MscL conformation is larger than that of TFE (Fig. 3).

The MD simulations show a clear difference between 5-FU and TFE at the extracellular side. The superposition of MscL (not treated), treated with 5-FU and TFE are shown in Fig. 1. The biggest change is observed for 5-FU, which occurs at the upper part of the TM2 helix (ILE40-Gly51) as well as the N-terminus helix. We chose to depict the extracellular diameter to quantify the extent of movement in the TM2 for all the conditions. We believe these changes are mostly reflective of change in the bilayer thickness and we do not expect to see any changes in the hydrophobic pore diameter.

**Figure 4:**
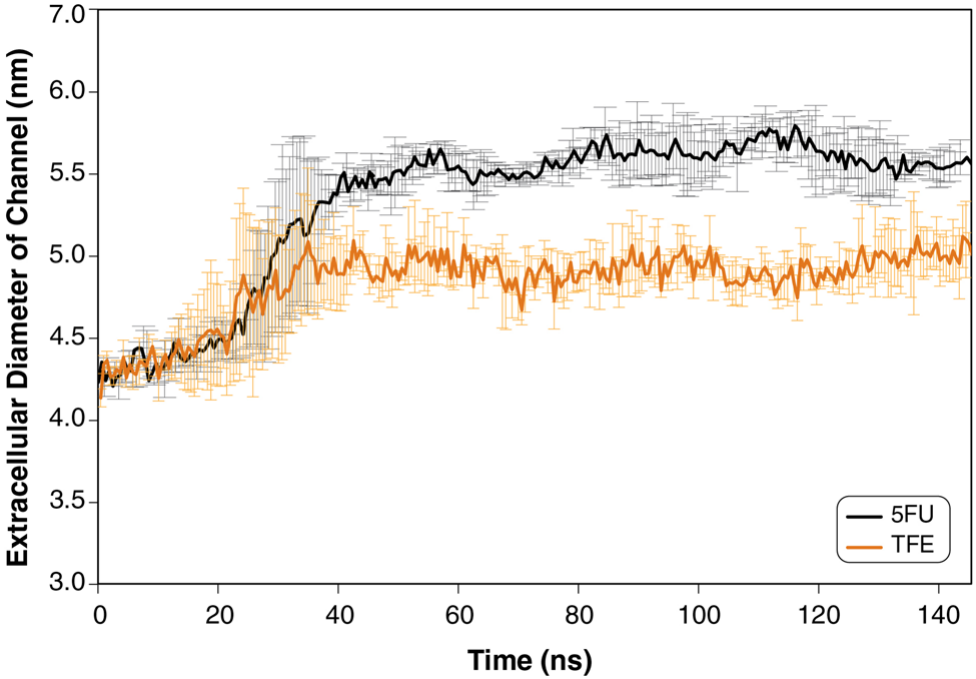
Effect of 5-FU and TFE on MscL conformation. Extracellular diameter of MscL increases when added 5-FU and TFE are added from the extracellular side. 5-FU exerted a larger effect on MscL conformation compared to TFE. Data represents mean ± SD of 3 replicate simulations.

As a result, incorporation of 5-FU into the bilayer changes the MscL extracellular diameter by ~ 30% (Fig. 4) priming the conformational change associated with the channel opening.

**Figure 5:**
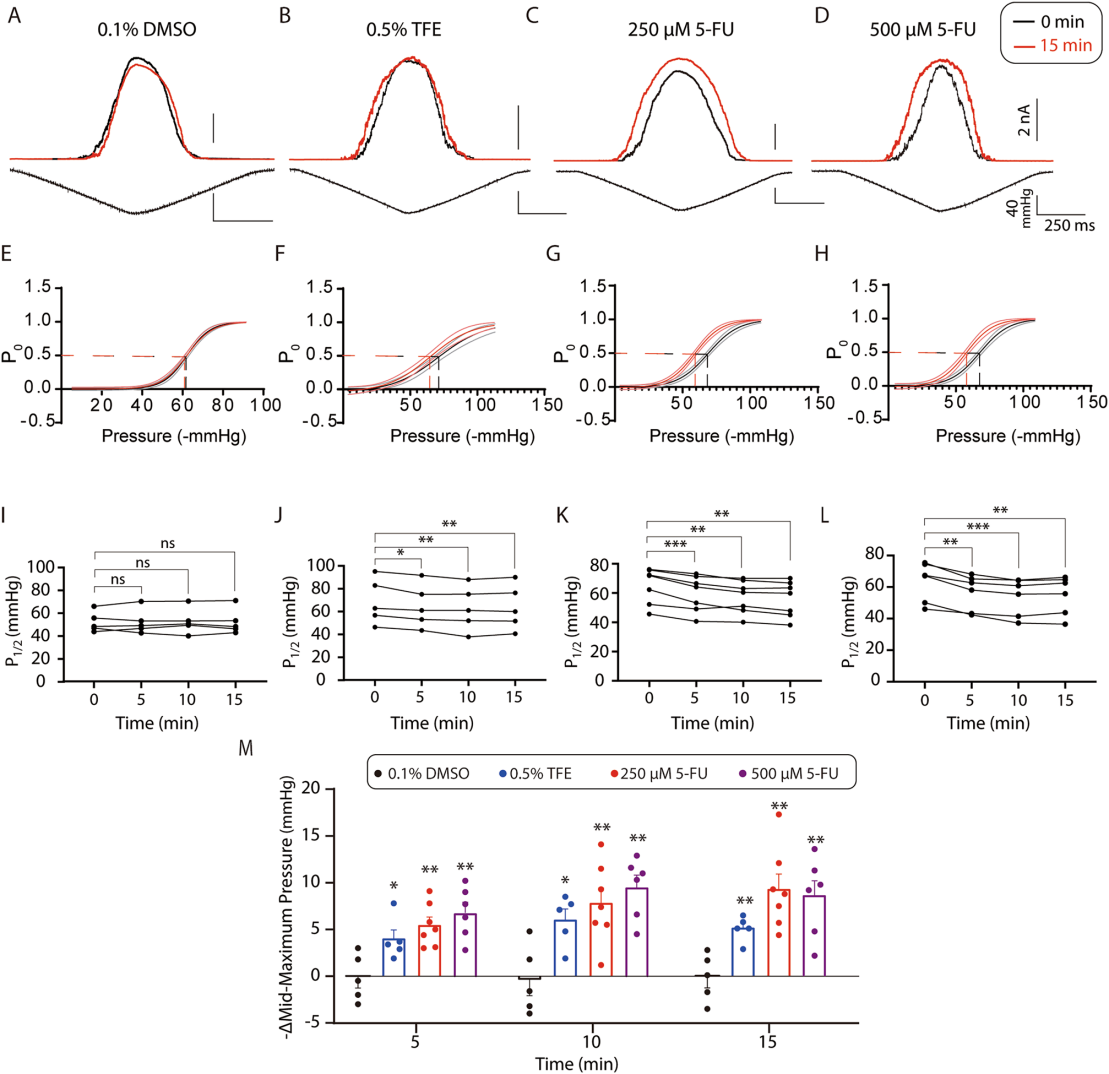
Effect of DMSO, TFE or 5-FU on gating of G22S-MscL reconstituted into liposomes. (**A**)–(**D**) Representative current traces showing activation of G22S-MscL channels upon application of pressure ramps recording under different conditions with and without application of 5-FU of TFE. The black traces indicate currents recorded before application of a drug, while the red traces indicate currents recorded at 15 min after exposure of the liposome patch to drugs. (**E**–**H**) Boltzmann distribution functions determined in the absence and the presence of each amphipath. 0.1% DMSO was used to dissolve 5-FU in the recording solution. Notice the parallel left-shift in negative pressure required for MscL-G22S gating in the presence of both TFE or 5-FU, but not in the DMSO control experiments. (**I**)–(**L**) Line charts showing the mid-maximum pressure (P_1/2_) of recordings in (**A**)–(**D**). Different from 0 min, for 0.1% DMSO, ns *P* = 0.9071, ns *P* = 0.8023, ns *P* = 0.9210 and n = 5; for 0.5% TFE, *P = 0.0177, **P = 0.0074, **P = 0.0011 and n = 5; for 250 µM of 5-FU, ***P = 0.0008, **P = 0.0029, **P = 0.0013 and n = 7; for 500 µM of 5-FU, **P = 0.0018, ***P = 0.0008, **P = 0.0044 and n = 6, at 5, 10 or 15 min after treatment. (M) Summary data of shifts of mid-maximum pressure. The shifts were calculated by subtracting mid-maximum pressure obtained at each time point from that obtained at 0 min. Different from DMSO control, for 0.5% TFE, *P = 0.0273, *P = 0.0133, **P = 0.0037 and n = 5; for 250 µM of 5-FU, **P = 0.0025, **P = 0.0058, **P = 0.0015 and n = 7; for 500 µM of 5-FU, **P = 0.0020, **P = 0.0010, **P = 0.0032 and n = 6, at 5, 10 or 15 min after treatment.

To test the predictions of the 5-FU and TFE effect on the lipid bilayer we recorded activity of the MscL channels reconstituted into liposomes made of SPL:POPE (70/30) (Fig. 5). We also recorded MscL activity in liposomes made of SPL:POPE (50/50) (SI Fig. 4) to mimic closer the bilayer properties in MD stimulations to the patch clamp experiments. Furthermore, to better detect the drug effects on the reconstituted MscL, we used the more tension-sensitive MscL mutant, MscL–G22S^13,57^. In this channel mutant, the mutation of Gly to Ser residue in the hydrophobic part of the channel pore results in a reduction of energy required for the channel activation. Importantly we note that it has been previously shown that MscL^58^ and MscS^17,18,59^ are oriented right-side-out when reconstituted using Dehydration/Rehydration method.

To estimate the change in mechanosensitivity of the MscL channel due to the effect of 5-FU and TFE on MscL-G22S, we measured the pressure applied to a patch pipette corresponding to half-activation of the MscL-G22S channels represented by the 50% open probability P_1/2_. We plotted the channel open probability data obtained at different negative pressures applied to the patch pipette and fitted them to Boltzmann distribution functions allowing us to determine P_1/2_ at different experimental conditions (Fig. 5). Since 5-FU had to be dissolved in DMSO before addition, we also measured the channel open probability in the presence of DMSO as a control (SI Fig. 5).

**Figure 6:**
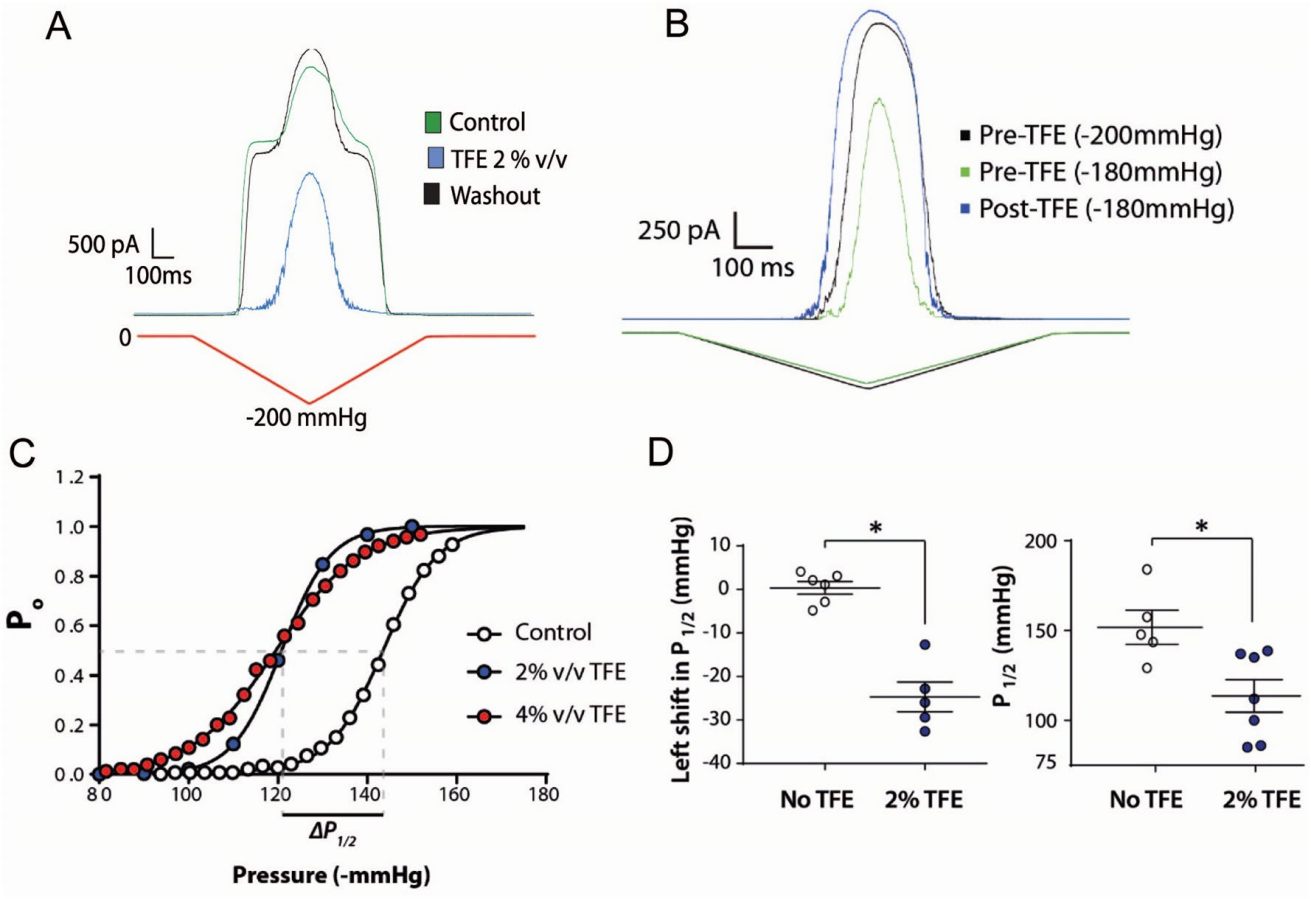
Differential effect of TFE on MscL and MscS expressed in MJF612 *E. coli* spheroplasts. (**A**) MscL and MscS are activated by application of a pressure ramp to an excised spheroplast patch (green and black trace). Addition of TFE from the cytoplasmic side reversibly abolishes MscS activity (blue trace showing only MscL activity). (**B**) Pressure ramps activating MscL channels in MJF612 spheroplast membranes pre and post addition of 2% v/v TFE. (**C**) Representative Boltzmann distribution functions of the MscL open probability in the absence and the presence of 0, 2 and 4% v/v TFE. (**D**) Quantification of the magnitude of the leftward shift in the P_1/2_ of MscL after 2% v/v TFE addition and quantification of the absolute change in the P_1/2_.

Furthermore, to investigate whether the effect of the amphipaths may differ depending on the type of MS channel and lipid composition, we examined the effect of TFE on MscS and MscL channels in bacterial spheroplasts (Fig. 6). Addition of TFE to the cytoplasmic side of the excised spheroplast patch not only robustly inhibits the activation of MscS but it also sensitizes MscL. The effect of TFE on MscS has previously been reported^60^ and the asymmetry of the response has been used as a pharmacological tool to study the direction with which MscS reconstitutes into liposomes^18,59^.

**Figure 7:**
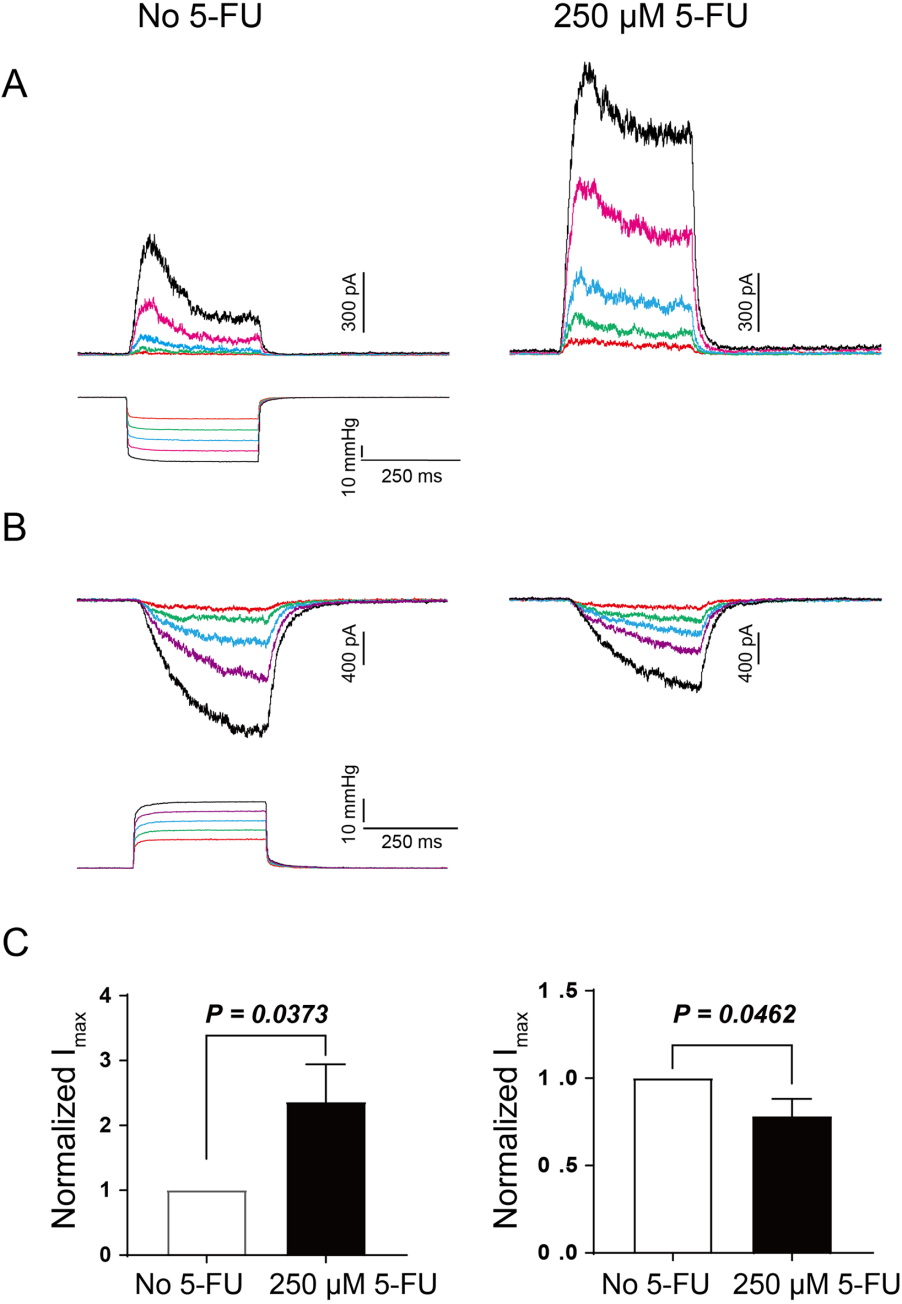
Effect of 5-FU on TREK-1 heterologously expressed in HEK293T cells. (**A**, **B**) 5-FU sensitizes TREK-1 from the cytoplasmic side (I/O), while it reduces the current from the opposite leaflet (O/O). (**C**) Normalized current before and after 5-FU, inside-out (left, n = 7, *p* = 0.0373) and outside-out (right, n = 8, *p* = 0.0462).

Eukaryotic MS channels including the two-pore domain type potassium channels TREK-1 and TRAAK, as well as the Piezo1 channel were shown to be activated by membrane tension similar to MscL and MscS channels^15^. To perform patch-clamp experiments with TREK-1 channels, they were expressed in HEK293T cells and recordings with and without 5-FU were carried out in inside-out and outside-out patches (Fig. 7). Opposite to MscS, TREK-1 channels were sensitized by 5-FU added from the cytoplasmic side and were inhibited by 5-FU added from the extracellular side. Similar results were obtained with TFE applied to TREK-1. In inside-out patches the current increased 10–15-fold when 2% TFE was added to the bath solution (SI Fig. 4). In outside-out patches the effect of TFE was the opposite causing a reduction in TREK-1 currents similar to the effects observed with externally applied 5-FU (SI Fig. 6).

## Discussion

Using molecular dynamics simulations and patch clamp recording, we investigated the effect of amphipathic compounds 5-FU used for cancer treatment and the anaesthetic TFE on MS channel activity. Overall, both drugs have significant effects on the physicochemical properties of the bilayer such as: bilayer thickness, lateral bilayer pressure profile, order parameter, and channel conformational changes. This is likely due to the differences in size and hydrophobicity between the two compounds similar to what was reported for the effects of chlorpromazine and trinitrophenol on the activity of MscS^23^.

**Figure 8:**
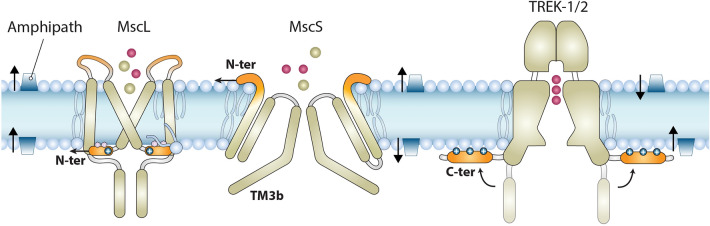
Diagram summarizing the effect of TFE and 5-FU on different types of MS channels. TFE (), sensitization (), desensitization ().

By examining the effect of both 5-FU and TFE on different MS channels gated according to the force-from-lipids principle, our data indicate that the shape and structural dynamics of the channel protein and the way it is anchored in the membrane bilayer determine how an amphipath would affect its mechanosensitivity and gating properties. As shown in Fig. 8 summarizing our results, TFE sensitizes MscL added from both bilayer leaflets. The effect of TFE on MscS was first examined by Akitake et al.^60^ who showed that cytoplasmic addition enhances inactivation and results in complete abolition of channel activity. In contrast from the extracellular/periplasmic side TFE sensitises MscS. Opposite to MscS, TREK-1 which is known to be affected by amphipathic molecules^61^ becomes sensitized by 5-FU and TFE added from the cytoplasmic monolayer and desensitized by TFE and 5-FU inserted into the outer monolayer. The sensitizing effect of TFE on TREK-1 may not be surprizing given that TFE, although not used as an anaesthetic in medical practice because of its toxicity, has activity similar to general anaesthetics which are known to potentiate TREK-1 channel activity^62^. Also the more pronounced effect of amphipaths on TREK-1 when added to the inner leaflet is consistent with the asymmetric structure and activation of TREK channels^63^.

The above discussion of leaflet specific effects of these amphipaths assumes that flip-flop of these molecules is negligible. Indeed, over our simulation time we see no examples of these amphipaths, or lipids for that matter, flipping between monolayers. However, we cannot exclude this possibility due to the relatively short simulation time course.

How do the asymmetric structures of MscS and TREK-1 align with the bilayer leaflet-dependent effect of TFE, 5-FU or any other amphipathic molecule on the activity of these two channels, whereas the amphipath effect on the MscL sensitisation by membrane tension is seemingly leaflet independent? The structure of both MscS and TREK-1 offer relatively simple explanations for the leaflet-dependent effect of the amphipaths. The N-terminal domain of MscS and C-terminal domain of TREK-1 are located on the extracellular and intracellular side of the bilayer, respectively, where the insertion of TFE and 5-FU sensitises the channels to membrane tension (Fig. 8). These structural domains were shown to control the gating of MscS^64^ and TREK-1 channels by mechanical force^65^. In the case of MscL, the insertion of the amphipaths into any of the two bilayer leaflets renders the channel more mechanosensitive. There are two possible reasons for this. First, the MscL structure is more symmetric with regard to the extracellular and intracellular membrane planes compared to either the MscS or the TREK-1 structure (Fig. 8). Second, the N-terminal cytoplasmic domain and the periplasmic loop of the channel play an essential role in MscL gating. The amphipathic N-terminal helix of MscL was shown to be crucial for coupling the channel to the membrane and activation by membrane tension as well as stabilization of its closed state^2,66^, whereas the periplasmic loop was shown to play a role in setting channel mechanosensitivity^67,68^. Consequently, insertion of amphipathic molecules into a bilayer leaflet from both sides of the membrane can sensitise MscL to mechanical force.

Previous work implicates the roles of lipid filled pockets in the gating of many mechanosensitive channels^2,14,52,54,69,70^. One alternate mechanism explaining amphipath mediated MS channel activation/sensitization is amphipaths displacing lipids in the pockets priming the channels for gating. However, we did not observe any insertion of the amphipaths in this study into lipid filled pockets or grooves in MscL but must note that our simulation time is short when compared to electrophysiology experiments and thus we cannot completely discount this possibility.

Concerning drug delivery systems^71–75^, liposomes provide a widely applicable method of drug encapsulation and administration. Given its size and comparably simple yet malleable structure as well as the very large pore of the open channel^76^, a genetically modified MscL channel, reconstituted into liposomes, has been considered for number of years as a potential nanovalve for triggered release of drugs. This study indicates that the presence of drugs, even in small doses, could have dramatic effects on the integrity and physical properties of liposomal carriers. This effect may be far more critical, especially when using protein channels as controllable nanovalves in smart drug delivery systems. We find that these drug—carrier interactions do not conform with the traditional picture of long-time retention of a drug within a hydrophobic core of the liposomal carrier. This issue highlights the need to further investigate the compatibility and durability of these drug-carrying compounds.

## Conclusion

Bilayer active amphipathic molecules generate membrane forces by one-sided membrane insertion, this differentially affects the activity of MS channels depending on the channel structure and structural dynamics. For instance, both TFE and 5-FU sensitized TREK-1 channels when added from the cytoplasmic side and desensitized TREK-1 when added to the extracellular side. At similar concentrations, compared to TFE, 5-FU had a more profound effect on bilayer mechanics such as the order parameter and bilayer pressure profile as well as on MS channel activity due to its larger and asymmetric size and depth of penetration in the membrane. Given that many physiologically active molecules such as PUFAs have amphipathic physico-chemical properties, our findings suggest that regulation of the activity of membrane proteins sensitive to mechanical stimuli by amphipaths may present a general regulatory mechanism in cells and tissues. Concerning liposomal drug delivery systems and the amphipathic character of many toxic drugs, our results suggest that this general mechanism may be exploited for sustained administration of liposome-encapsulated drugs.

## Supplementary Information

Supplementary Information.

## Data Availability

The datasets used and/or analysed during the current study available from the corresponding author on reasonable request.
