# Supplementary material for: Asymmetric effects of amphipathic molecules on mechanosensitive channels
Source: Sci Rep. 2022 Jun 15;12:9976. doi: 10.1038/s41598-022-14446-w (PMC9200802; doi:10.1038/s41598-022-14446-w)
Supplement: Supplementary file 1 — Supplementary Information. [file 41598_2022_14446_MOESM1_ESM.docx]

**SUPPLEMENTARY INFORMATION**


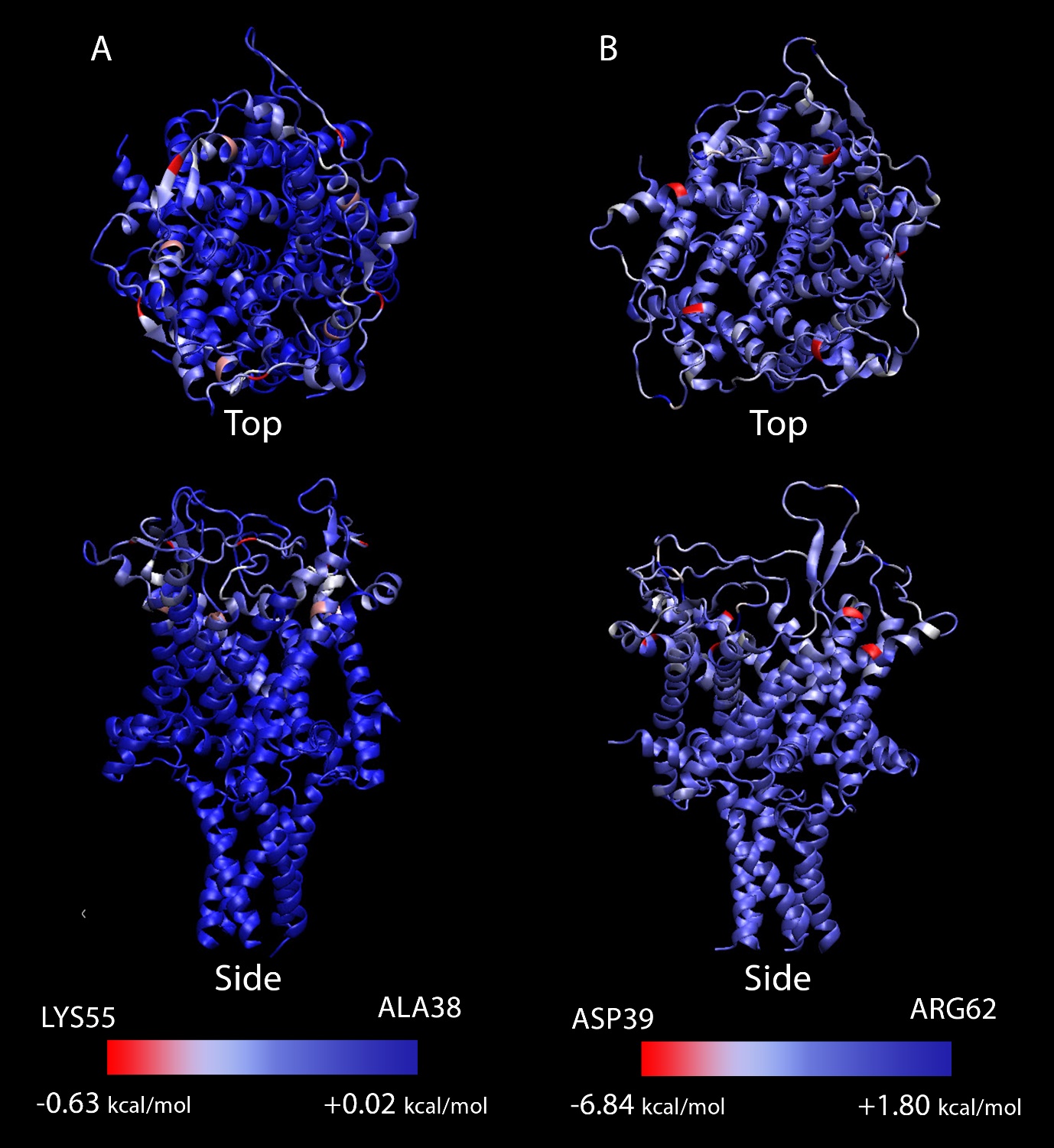


**SI Figure 1. The residues directly interacting with TFE (A) and 5-FU (B).** The interaction energy varies from +0.02 kcal/mol (ALA38) to -0.63 (LYS55) for TFE and +1.80 kcal/mol (ARG62) to -6.84 (ASP39) for the 5-FU system. These interaction values are per MscL subunit.


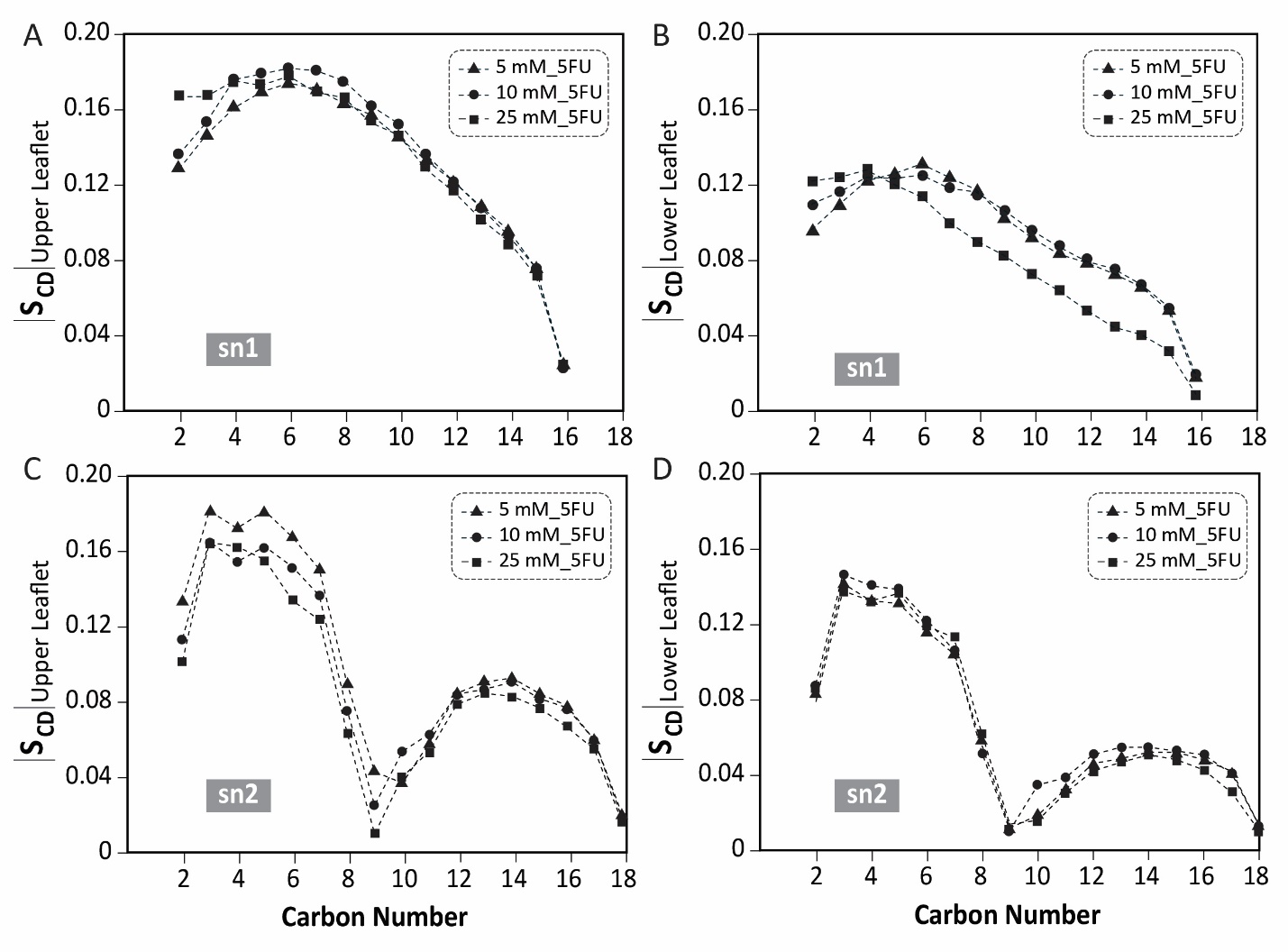
 **SI Figure 2. Effect 5-FU on sn1 and sn2 order parameters at three different concentrations.** As the concentration of 5-FU increases, the sn1 order parameter for the upper leaflet (the side containing the drug) increases while sn2 decreases. For the lower leaflet, the sn1 parameter decreases with boosting the 5-FU concentration, while Sn2 parameter remains almost unchanged.


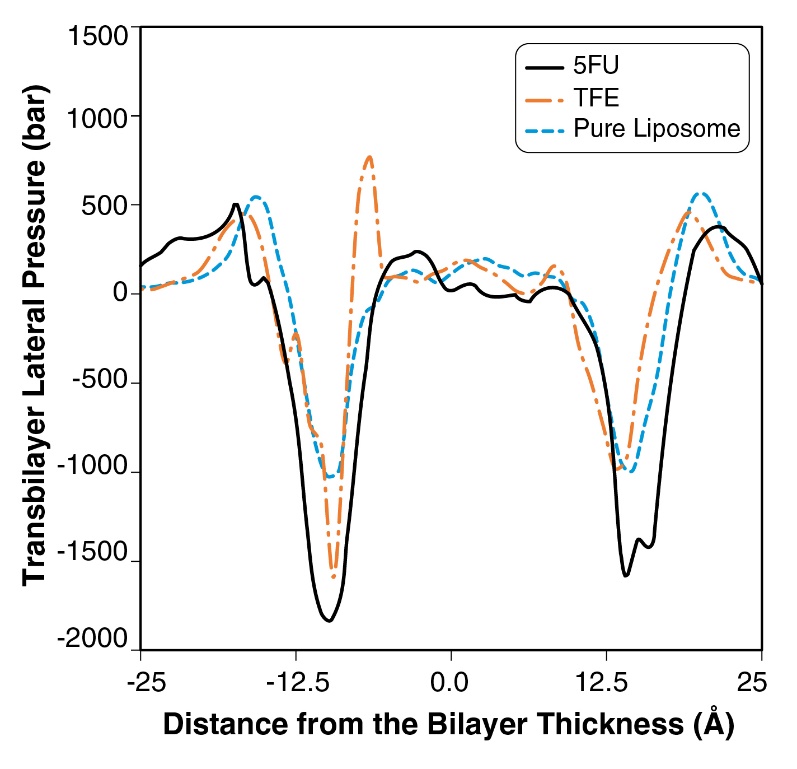


**SI Figure 3. Effect of 5-FU and TFE on the lateral pressure profile of the lipid bilayer.** The presence of the 5-FU and TFE molecules introduced significant changes in surface tension and symmetry of the transbilayer pressure profile. The TFE effect is largest around the lipid head groups of the leaflet of which it is added. Whereas 5-FU affects both leaflets at high concentrations.


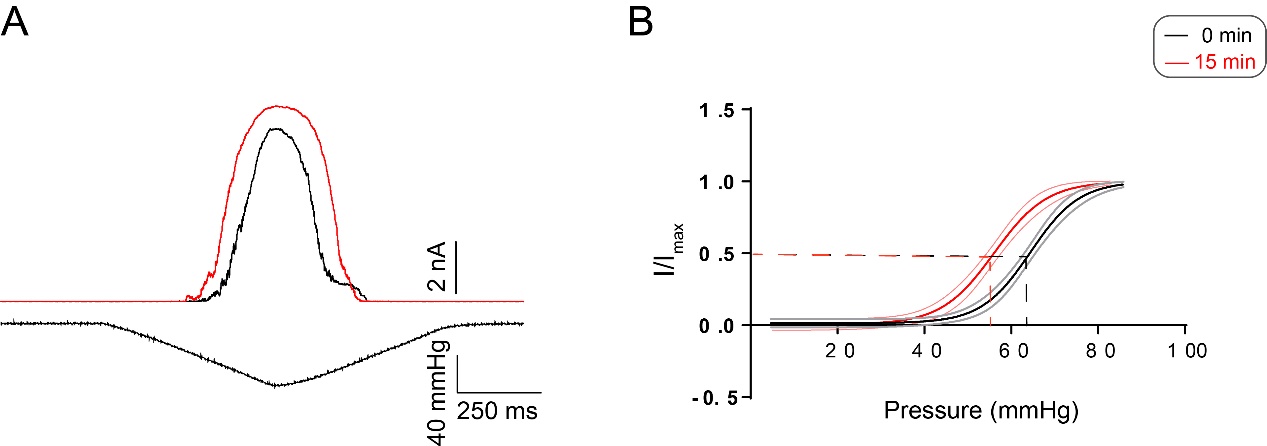


**SI Figure 4. Effect of 500 µM 5-FU on the open probability of MscL-G22S reconstituted in SPL:POPE 1:1.** Boltzmann distribution functions determined in the absence and the presence of 5-FU. Estimated P_1/2_ in mmHg are: 64 and 56 for 0 or 15 minutes after adding 500 µM of 5-FU. n = 3 independent recordings.


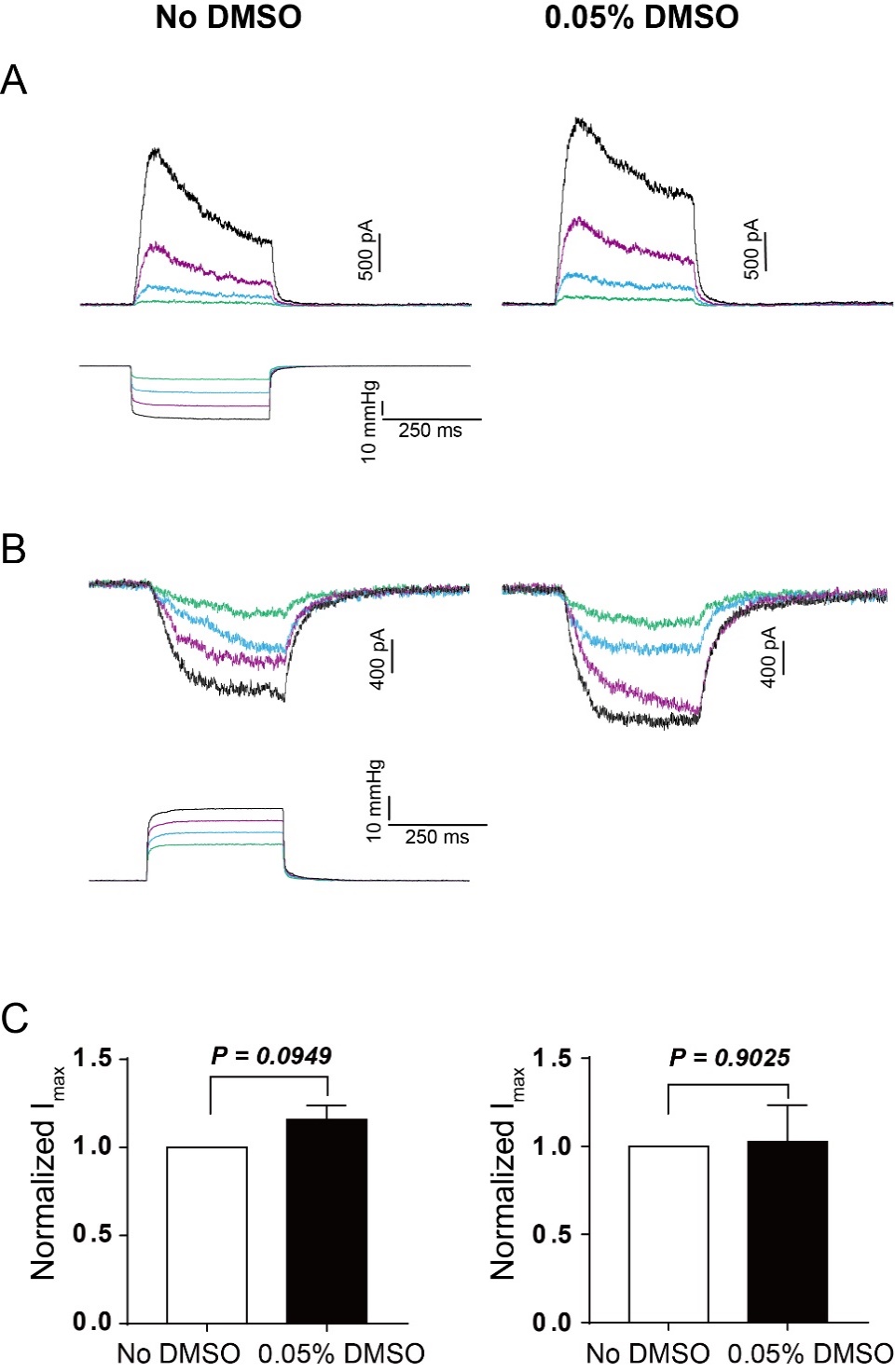


**SI Figure 5. Effect of 0.05% DMSO on TREK-1 heterologously expressed in HEK293T cells**. **(A, B)** 0.05% DMSO does not have significant effect on TREK-1 from both the cytoplasmic side (I/O) or the opposite leaflet (O/O). **(C)** Normalized current before and after DMSO, inside-out (left, n = 4, p = 0.0949) and outside-out (right, n = 5, p = 0.9025).


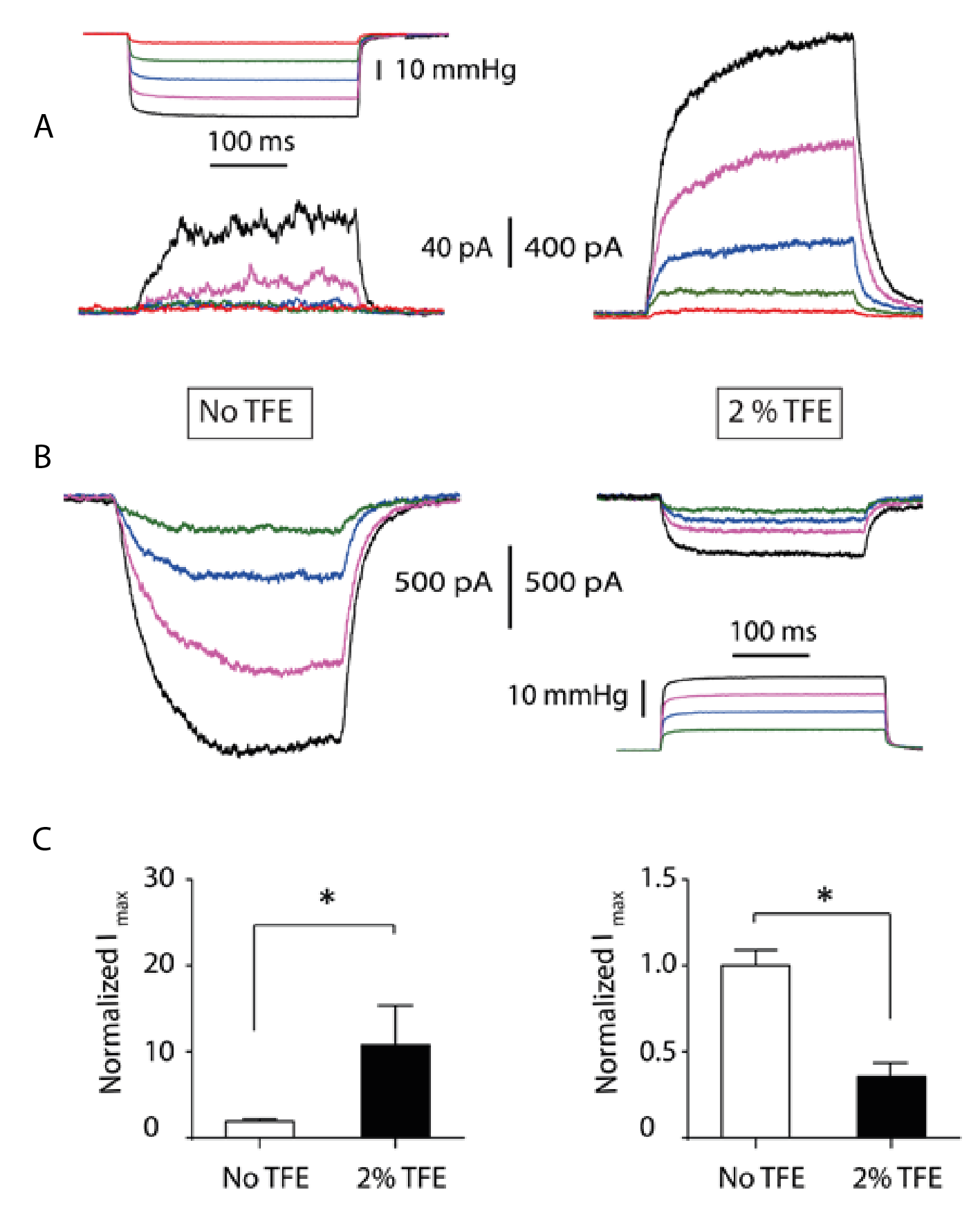


**SI Figure 6. TFE potentiates TREK-1 activity from the cytoplasmic side and inhibits it from the extracellular side. (A)** Pressure steps applied to TREK-1 channels in an excised inside-out patch from HEK293T cells +30 mV. **(B)** Pressure steps applied to TREK-1 channels in an excised outside-out patch from HEK293T cells -30 mV before and after addition of 2% TFE. (C) Quantification of the effect on maximal TREK-1 currents after TFE addition in inside-out (left panel) and outside-out (right panel) configurations. Data represents mean ± SEM, n=6-7.

Table S1. Drug-Protein Interaction Energy for TFE and 5-FU systems.

| **Drug** | **Interaction Energy (kcal/mol)** |
| --- | --- |
| TFE | +0.02 (**ALA**38) to -0.63 (**LYS**55) |
| 5-FU | +1.80 (**ARG**62) to -6.84 (**ASP**39) |
